# Are treatment plans optimized on the basis of acuros XB dose calculation robust against anatomic changes during online adaptive radiotherapy for lung cancer regarding dose homogeneity?

**DOI:** 10.1186/s13014-025-02656-1

**Published:** 2025-05-15

**Authors:** Khouya Aymane, Santiago Alina, Ringbaek Toke, Guberina Nika, Guberina Maja, Gauler Thomas, Lübcke Wolfgang, Zylka Waldemar, Pöttgen Christoph, Stuschke Martin

**Affiliations:** 1 Department of Radiotherapy, West German Cancer Center, University Hospital Essen, Hufelandstraße 55, 45147 Essen, Germany; 2 German Cancer Consortium (DKTK), Partner Site University Hospital Essen, Essen, Germany; 3 Faculty of Physical Engineering, Westphalian University, Campus Gelsenkirchen, Neidenburger Str. 43, 45897 Gelsenkirchen, Germany

## Abstract

**Introduction:**

The Acuros XB dose calculation algorithm implements advanced modelling of lateral electron transport, making dose distributions sensitive to density changes between source and subsequent CT. The aim of this study was to analyse the robustness of dose distribution in the central bronchial wall (CBW) of treatment plans from lung cancer patients treated with adaptive radiotherapy.

**Material and methods:**

IMRT or VMAT plans from patients with locally advanced lung cancer from a prospective registry cohort were analysed, who received definitive radiotherapy in surface-guided inspiratory breath-hold on the Ethos™ closed-bore linac, equipped with the HyperSight™ cone beam CT (CBCT). Dose homogeneity of the scheduled plans, optimized on planning CT (CTplan), was verified on the initial CBCT of a dose fraction (CBCT1). The adaptive plans were verified on a subsequent post-adaptation CBCT (CBCT2) of the same dose fraction. A predictive model was built for maximum dose (Dmax) in CBW in dependence on plan sensitivity in the central bronchial air lumen overlapping the planning target volume (CBAL_PTV_) to water override (WOR) of the air lumen.

**Results:**

Ninety-one dose-fractions from 10 patients were analysed. Dmax values in the CBW of the scheduled plans showed over all significant inter-fractional increases from CTplan to subsequent CBCT1 (*p* < 0.0001, Wilcoxon test, stratified by patient) with significant heterogeneity between patients (*p* < 0.0001, Kruskal-Wallis Test). The median Dmax increase per dose fraction was 2.15% (-3.15 − 19.30%). Reducing the PTV overlap of scheduled plans with CBAL led to lower inter-fractional Dmax increases in CBW (*p* < 0.0001, signed rank test). Dose accumulation showed, that Dmax and D1cc values in CBW over the treatment course stayed in all patients below 110.5% and 107.5% and that the equivalent uniform dose in CTV around the CBW stayed > 95% for scheduled plans. A predictive model showed the dependence of inter-fractional Dmax increases in CBW of scheduled plans on an interaction between plan sensitivity on CTplan to WOR in CBAL_PTV_ and density change at the Dmax point in CBCT1 between CTplan and CBCT1 (*p* < 0.0001, t-test). Intra-fractional Dmax increases of adaptive plans in CBW amounted to only 20% +/- 1.1% of the inter-fractional increases of scheduled plans, as intra-fractional deformations were smaller than inter-fractional (*p* < 0.0001, signed rank test).

**Conclusion:**

Dose homogeneity in CBW of Ethos plans were found sufficiently robust against intra-fractional deformations during course of online adaptive radiotherapy. Plan sensitivity to anatomic changes can be detected and controlled on the planning CT by the WOR of air in CBAL_PTV_.

**Supplementary Information:**

The online version contains supplementary material available at 10.1186/s13014-025-02656-1.

## Introduction

Adaptive radiotherapy (ART) can reduce normal tissue exposure in patients with locally advanced lung cancer. This is achieved by adjusting the target volume based on tumor shrinkage or anatomical changes observed during treatment. Such adaptations are guided by mid-treatment course, weekly replanning CT, or a triggered replanning CT based on anatomic changes discovered in daily Cone-beam CT(CBCT) for image guidance [1–3]. Online adaptive treatment plans can further reduce dose to surrounding normal tissues, particularly when intra-fractional motion is smaller than inter-fractional motion and significant anatomical changes occur between the baseline planning CT (CTplan) and daily anatomy [4–9]. In this context, online adaptive radiotherapy (oART) is especially valuable for protecting the central bronchial wall from overdosage during ablative radiotherapy treatment for ultracentral lung tumors [7].

The proximal or central bronchial structures, i.e. trachea, main bronchi, intermediate bronchus and lobar bronchi up to their first bifurcation, can express several adverse effects from radiotherapy. These include bleeding, stenosis, or fistula formation. Studies on stereotactic ablative radiotherapy (SABR) for central lung tumours estimate that the total equivalent dose of 2 Gy per fraction associated with a 5–10% risk of severe side effects to the central bronchial tree ranges between 65 and 85 Gy [10–12]. Similarly, studies on hypo-fractionated dose escalated radiotherapy showed increased incidence of higher grade lung toxicities in dependence on dose-volume parameters from the central bronchial tree [13, 14].

In low-density air cavities, the lateral range of secondary electrons increases. As a result, more electrons scatter outward than inward at the boundaries of small photon fields [15, 16]. It has been shown, that dose reduction in a low density cavity within a higher density medium increases as the distance to the field edge decreases, if the latter is smaller than the lateral range of electrons [17–19]. The Acuros XB algorithm, a linear Boltzmann transport equation solver, models the macroscopic behaviour of neutral and charged particles on an adaptive cartesian grid [20]. Older algorithms, such as the anisotropic analytical algorithm (AAA), which applies density scaling of dose kernels for heterogeneous media, have been shown to overestimate doses near air-tissue interfaces. It was observed that Acuros XB algorithm can predict doses at air-water interfaces with comparable precision as Monte-Carlo Methods and predicted lower doses in air and more severe secondary dose build-up beyond air than AAA algorithm [21–23]. Irradiation from multiple field directions can significantly reduce inaccuracies in treatment plans calculated by both the Acuros XB and AAA algorithms [23].

When using Acuros XB optimizing the dose distribution to achieve a homogeneous dose coverage of the planning target volume that contains voxels with varying CT numbers tends to increase fluence in air cavities at the boundaries of the high-dose region. This compensates for electron disequilibrium effects but can lead to increased plan sensitivity to anatomical changes. If air cavities move between the planning CT and subsequent CTs, hotspots may occur. The central bronchial tree, for instance, exhibits substantial breathing motion, with median amplitudes of 2.6 mm, 2.5 mm, and 5.2 mm in the mediolateral (ML), anteroposterior (AP), and cranio-caudal (CC) directions, respectively [24].

In patients with locally advanced lung cancer, large target volumes, such as those encompassing involved mediastinal lymph nodes and the primary lung tumour can deform from daily due to tumour shrinkage, different breathing patterns, set-up errors, or the occurrence of new pulmonary infiltrates. Voluntary breath-hold techniques are an appropriate method for reducing breathing motion during beam-on time. Due to expansion of the lower lobes, mean lung dose can be reduced for upper-lobe and centrally located lung cancers [25]. PTV margins to account for inter- and intra-fractional positional errors can be kept below 5 mm for small targets using surface-guided deep inspiration breath-hold for cooperative patients [26, 27]. Closed bore, fast rotating linear accelerators can deliver a Volumetric Modulated Arc Therapy (VMAT) or multifield Intensity Modulated Radiation Therapy (IMRT) within 150 s, further improving the reproducibility of radiotherapy in deep inspiration [28].

The first generation of the Ethos treatment system for oART used a synthetic CT for optimizing adaptive treatment plans by deforming the CTplan on the CBCT of the day [29]. However, synthetic CTs have limitations when large anatomical density changes occur between the source and subsequent CTs, such as new atelectasis that cannot adequately modelled by elastic deformation [30]. Wegener et al. demonstrated in phantoms that density changes in lung accompanied by shrinking peripheral lung tumors from 3 cm to 1 cm in diameter can lead to errors of up to -16% in the estimated mean target dose when calculated on a synthetic CT by the Ethos treatment planning system [31]. Recent advancements in CBCT technology now enable fast CBCT acquisition within 6 s, with reconstruction using advanced iterative algorithms featuring advanced scatter correction. The HyperSight™ CBCT system, integrated into the Ethos therapy system, provides geometric and CT number accuracy comparable to that of spiral fan-beam CT, making it suitable for treatment planning [32–34]. The gamma passing rates (1%/1 mm) of VMAT or IMRT treatment plans for a lung tumor in an Alderson Phantom were > 98% when calculated on HyperSight CBCT compared to spiral fan-beam CT in the dose region above 50% of the prescribed dose [35]. Acquiring HyperSight CBCT in inspiratory breath-hold can avoid motion artifacts that are present when acquisition is performed during free breathing [36].

The aim of the present study is to analyse the robustness of treatment plans optimized in Ethos based on the Acuros dose calculation algorithm. Specifically, we assessed how these plans maintain homogeneous and conformal dose coverage of the target volume in patients with inoperable, locally advanced non-small cell lung cancer (NSCLC), despite density changes caused by inter- and intra-fractional motion. We analyzed dose homogeneity in two regions: the central bronchial wall and in the target volume within 2 cm around the central bronchial tree. This evaluation included both scheduled plans, recalculated on HyperSight CBCTs across different fractions, and adaptive plans, calculated on the initial CBCT (before adaptation) and on the verification CBCT (after adaptation) for each treatment fraction. The study was conducted on consecutive patients enrolled in a prospective clinical registry.

## Materials and methods

### Patient cohort

Ten consecutive patients recruited in a prospective registry trial for patients with Non-Small Cell Lung Cancer (NSCLC) underwent ART using the Ethos™ therapy system (Varian Medical Systems, Palo Alto, CA, V.1.0) equipped with a Hypersight™ CBCT and the Identify surface guidance system (Varian Medical Systems, Palo Alto, CA, V.3.0). Treatment was performed between 10.2023 and 07.2024. Each patient underwent initial planning spiral fan beam computed tomography scan (CTplan) in deep inspiration breath hold, reconstructed with 1.5–2.0 mm slice thickness, followed by daily initial CBCT imaging at the beginning of each treatment fraction (CBCT1). A verification CBCT was acquired after plan adaptation immediately before treatment delivery (CBCT2). If necessary, an additional translational coach shift could be applied according to a rigid match of CBCT2 with CBCT1. The data exported from Ethos consists of CTplan, CBCT1, CBCT2, the scheduled plan representing an IGRT treatment and the adaptive Plan. The rigid clinical match between CTplan and CBCT1 was reproduced in MIM Maestro (Cleveland, OH, USA, MIM Software Inc, version 7.3.2). Here we created a point-based match between the plan Isocentre of the original plan on the CTplan and the plan Isocentre of the Scheduled Plan on CBCT1. This match is referenced to as (REG1). The clinical match between CBCT1 and CBCT2 is labelled as (REG2). Power analysis using the procedure G*power shows, that a sample size of 10 patients is sufficient to detect an effect size, characterized by coefficient of determination (R^2^) of 0.5 with power of 0.88 at alpha error probability of 0.05 using a one tailed t-test [37]. In addition, the sample size increases to 91 for analysis on predictors from each dose fractions as 91 Hypersight cone beam CT (CBCT) before and after dose adaptation were available and scheduled plans as well as the adapted plans on the preadapted were recalculated on each of them. All patients gave written informed consent before the study entry. This study was approved by the Ethics Committee of the University Duisburg Essen (18-8364-BO) and was registered at clinicalTrials.gov (NCT06222190).

### Parameter labelling and definition

Organs at risk and structures analysed for the present study were the central or proximal bronchial tree (CBT), defined as the trachea, the right and left main bronchi, the intermediate bronchus and the lobar bronchi up to their first bifurcation [38]. The central bronchial air lumen (CBAL) was identified as the content of the CBT with a density below − 600 HU. The region where the CBAL overlapped with the PTV was designated as CBAL_PTV_. The central bronchial wall (CBW) was defined as the contour of the CBT excluding the CBAL, with an outer margin of 8 mm. The CTV was contoured without the CBAL, while the central CTV (cCTV) was defined as the portion of the CTV within 2 cm around the CBT, also termed as the no-fly zone in stereotactic ablative radiotherapy of central lung cancers [39].

All parameters were labelled according to a consistent nomenclature to ensure their unique designation describing their derivation. Each parameter label consists of a short label for the considered derived physical quantity, followed by the structure from which the parameter was derived, followed by one or two CT studies combined with a forward slash in which the structure was contoured, and which were used for dose calculation, followed by the treatment plan from which a dosimetric characteristic was derived from. These four items were connected by an underscore character. Examples for short labels of derived quantities are Dmax, DeltaDmax, DHU_CBCT1/CTplan_@DmaxP. Dmax is the maximum dose in the assigned structure, DeltaDmax is the dose difference between the independent Dmax doses in the assigned structure on the first and second assigned CT, the first might be modified by water override of CBAL (+ WOR). DHU_CBCT1/CTplan_@DmaxP is the short label for the Houndsfield Unit difference between CBCT1 and CTplan coregistered by the clinical match at the Dmax point in the following structure. *DeltaDmax_CBW_CBCT1/CTplan_S1/2* reads as Dmax in CBW on CBCT1 minus Dmax in CBW on CTplan calculated by the scheduled plan according to both method 1 and 2. The treatment plans used here are defined in the following section as well as the abbreviations used for them.

### Treatment planning

All adaptive and scheduled plan optimization was performed in the Ethos™ therapy system using a goal list as described below. Translation of a goal list into objective function is automatically performed by the Ethos intelligent Optimization engine (IOE) without interference of the user [40]. The clinical plans were optimized using method 1 for the first 5 patients and method 2 thereafter. The goal list for method 1 required a D98% of the clinical target volume (CTV) ≥ 98% with priority 1 (P1), D98% of the planning target volume (PTV) ≥ 95% with P1 and Dmax ≤ 110% with P2. Additional constraints were set for organs at risk (OAR), such as the body (Dmax ≤ 105%, P2), oesophagus (Dmax < 104%), heart (Dmean at per patient achievable levels from the institution’s RapidPlan knowledge-based lung cancer planning model), left and right lung (Dmean < 18 Gy) as well as the spinal cord (Dmax at levels < 67% of the prescribed dose). For all OAR the priority chosen was P2. In contrast, the goal list for the PTV according to method 2 was adjusted to a less stringent constraint from D98% *≥* 95% to D95% ≥ 95% at P1 while the coverage of the CTV was maintained to D98% *≥* 98%. All other OAR constraints from method 1 were maintained, and an additional constraint was introduced for the central bronchial tree (CBT), limiting Dmax below 110% with priority 1.

With method 2, the PTV was derived from the clinical CTV adding an outer margin of 5 mm, that could overlap the CBAL, whereas the clinical CTV did not overlap with the CBAL (Suppl Fig. 3b and 4b). With method 1, the clinical CTV was unified with a CTV-supplement within CBAL. The CTV-supplement contained the portion of the CBAL that was surrounded on all sides by the CTV. In axial CT slices, where the borders of the clinical CTV abutted but not fully surrounded the CBAL, the contour of the CTV-supplement consisted of the abutting CTV borders and closed them by a straight line through the CBAL. An example for the CTV-supplement is shown in Suppl Fig. 4a. The PTV was generated from the supplement CTV by applying a uniform outer margin of 5 mm. An example for the CTV-supplement is shown in Suppl Fig. 4a. For patients treated with a clinical plan according to method 1, an additional plan according to method 2 was created retrospectively for this study. Vice versa, a plan according to method 1 was retrospectively created for patients who were treated with a plan according to method 2. In addition, a third method was retrospectively evaluated with the same goal list and CTV as method 2, but the overlap of the PTV with CBAL was limited to a margin of 3 mm (Suppl Fig. 2c). Short labels for the treatment plans according to the different methods were S1, S2 and S3, which refer to the scheduled plans according to method 1, 2, and 3, while Sclinical represents the scheduled plan according to the method clinically used for the respective patient. Adaptive plans optimized by the method used in the clinic are denoted by “Adapt”.

All treatment plans optimized in the Ethos system were recalculated in Eclipse (Varian Medical Systems, Palo Alto, CA, Version 16.00.00) using two distinct dose calculation algorithms: Acuros XB (Version AXB_16.1) and the Anisotropic Analytical Algorithm (AAA, Version AAA_16.1). The comparison between Ethos Acuros XB and Eclipse Acuros XB dose calculation algorithms was conducted in the CBW, the CBAL_PTV_, CBAL and CTV, using a gamma index analysis in MIM Maestro, applying a 2%/2 mm criterion. Percent depth dose (PDD) curves along the central beam axis of a 6 MV, 10 × 10 cm² photon field, as well as lateral dose profiles through the center of a rectangular air cavity within a digital water-air phantom, were calculated using Acuros XB in Eclipse. These results were compared to published measured values and Monte Carlo simulations for quality assurance, demonstrating good agreement within 2% of the maximum depth dose value [41].

### Plan robustness evaluation in CBW against density changes caused by anatomic deformations

The co-primary end points of the present study are the Dmax or DeltaDmax_CBW_subsequent CT/source CT_S1/2 values or the respective parameters using the adaptive plans. These parameters were used to describe the plan robustness against density changes from source to subsequent CT near the air cavity of the CBT. In addition, sensitivity of the dose distribution from a plan calculated with or without WOR of CBAL in the source CT was characterized by the DeltaDmax values within CBAL_PTV_.

### Plan recalculation on hypersight-CBCT

The effect of anatomic changes on the dose distribution by a given plan optimized on the source CT was evaluated on a subsequent CT. In the IGRT scenario, the source CT refers to the CTplan, and the subsequent CT corresponds to Hypersight CBCT1. In adaptive scenarios, the source CT is Hypersight CBCT1, and the subsequent CT is Hypersight CBCT2. The plans were recalculated in Eclipse with Acuros XB and applied to all CBCT1 scans by using the clinical match REG1. For the adaptive workflow, we used the adaptive plans generated in Ethos and recalculated those in Eclipse on CBCT1 and CBCT2. A dedicated CT number calibration curve was generated for the Hypersight CBCT scans using the Electron density phantom model Gammex RMI 467. HyperSight scans were acquired in the Thorax acquisition mode using iterative reconstruction and advanced scatter correction.

Dmax values within the CBW, the primary end point of this study, were recalculated with SciMoCa™ Monte Carlo for plan verification [42] using ProSoma software (Darmstadt, Germany, MedCom, Version 4.2.416) for each fraction with the highest Dmax in CBW on CBCT1 of each patient.

### Inter- and intra-fractional anatomical deformations

The anatomical shift of the CBW was assessed by comparing its position on the source and subsequent CTs in relation to the scheduled plan. First, both scans were aligned using the clinical rigid registration provided by the Ethos system. To evaluate inter- or intra-fractional anatomical deformations of the patient by assuming the CBW as a rigid structure, an additional contour-based rigid registration was performed in MIM Maestro software between the CBT in the source and subsequent CTs. The length of the displacement vector||CBTree|| and its DICOM coordinates in x (left lateral - right lateral), y (posterior - anterior) and z (superior -inferior) was subsequently computed as the subtraction of this contour-based CBT alignment and the clinical co-registration vector between both CT-studies. The short label used for the length of the rigid displacement vector used is Shift_source CT/subsequent_ CT_||CBTree||.

Considering also deformations of the CBW, the deformation vector at the Dmax location in CBW of the subsequent CT was determined using a hybrid density and structure based deformable image registration between CBCT1 and CTplan for inter-fractional deformations and from CBCT2 to CBCT1 to assess intra-fractional deformations, performed with MIM Maestro. The structure use for this deformation was the CBT. The length of the deformation vector was sampled from a sphere with a 1 mm radius around the Dmax point in the CBW of the subsequent CT. The spatial variation at the Dmax location was characterized by computing the mean distance to agreement (MDA) between the sphere copied from the subsequent to the source CT, both linked by the clinical registration, or a sphere transferred by the deformable image registration. The short label for the length of the deformation vector is Shift_source CT/subsequent CT_@DmaxP_CBW_subsequent CT_Planx.

The diffplanV10CBAL is the volume in CBAL with a dose increase by more than 10% of the prescribed dose obtained from the dose difference-distribution between a Plan x on the source CT calculated with or without water override of the CBAL. V10_CBAL_ is the isodose volume within CBAL, obtained from the dose difference-distribution between a Plan x on the source CT calculated with or without water override of CBAL. It represents the volume within CBAL with a dose increase of more than 10% of the prescribed dose due to WOR. To assess the spatial relationship between the Dmax location in CBW on the subsequent-CTs and the *V10*_*CBAL*_ volume on the source CT, we calculated the Hausdorff distance between both. For this calculation, the Dmax location on the subsequent CT was transferred to source CT using deformable image registration. The short label used for this distance was HD(diffplanV10_CBAL,_ DmaxP_CBW_subsequent CT)_Source CT_Planx.

### Dose accumulation and spatial analysis of Dmax positions

Dose distributions were accumulated using MIM. The accumulated dose distributions in CBW or in cCTV were generated using a CBW- or cCTV-based hybrid deformations, aligning the subsequent CT scan with the source CT to ensure precise registration of the respective structures. The CTV on CBCT2 was automatically generated using a hybrid deformation algorithm that propagated the CTV from CBCT1 to CBCT2 in MIM. All CTV and contoured normal tissue structures on CBCT2 were reviewed by an expert radiation oncologist for lung cancer and corrected if necessary. All recalculated dose distributions on subsequent CT studies were mapped twice onto the source CT using both the CBW and the cCTV based elastic deformations. From the CBW-based accumulation, Dmax and the minimum dose within the 1 cubic centimetre at highest dose (D1cc) within the CBW were extracted, characterizing high-dose regions within the CBW. In addition, the generalized equivalent uniform dose (EUD) was calculated for the cCTV, with a tissue parameter of a=-20 for aggressive tumors [43].

### Statistical analysis

Descriptive statistical analysis was conducted using SAS software version 9.4, SAS/STAT15.1 (SAS-Institute, Cary, NC). Procedure NPAR1WAY was used to perform the stratified Wilcoxon or Kruskal Wallis tests. The stratified Wilcoxon test combines the stratum level rank sum statistic of Wilcoxon scores of a characteristic from different anatomical scenarios per patient, i.e. from CTplan versus CBCT1 or from CBCT2 versus CBCT1. It analyses intra-patient differences between these anatomic scenarios over all patients. The Kruskal Wallis test uses Wilcoxon scores of a characteristic determined over all patients to detect differences between patients. Procedure Univariate to perform the signed rank tests. The linear predictive model was build using the procedure GLM, modelling the dependent variable, e.g. Dmax values in CBW by the scheduled plans (Fig. 2a) or DeltaDmax in CBW by the scheduled plan (Fig. 2c), in dependence on density changes in Hounsfield Units [HU] between CBCT1 and CTplan in plan coordinates at this point with maximum dose in the CBW on CBCT1 (ΔHU_CBCT2/CBCT_@DmaxP_CBW_CBCT2_S1/2 [HU]) as one main effect and on the parameter for plan sensitivity on CTplan for density changes in the CBAL with or without WOR (DeltaDmax_CBAL_PTV__CTplan *±* WOR/CTplan_S1/2) as the second main effect as well as the multiplicative interaction effect of both. In addition, an intercept effect was allowed. To assess the internal validity of the model and to reduce the generalisation error, fivefold cross-validation was used. The dataset was randomly split into five disjunctive subsets at equal probability that on observation falls in one of the five subgroups. The model was built from scratch on four subsets and the leave-out subset was scored by the fixed model from the training set so that the model is independent from the leave out data. This was repeated for all leave-out subsets, ensuring that the entire dataset was evaluated by a model generated on independent data. Cross validation was used to assess how the results will generalize to an independent data set [44]. 3D graphics were plotted using the procedure g3d. A correlation matrix between Dmax values in CBW was analysed using hierarchical cluster analysis (Procedure VARCLUS, SAS). The variables from the same cluster show a higher correlation with each other but have a low correlation with any other cluster. The procedure tries to maximize the variance explained by the clusters, summed over all the clusters. A dendrogram of hierarchical clusters was plotted.

## Results

Ten patients with inoperable locally advanced NSCLC from a prospective registry trial were treated with online adaptive radiotherapy in deep inspiratory breath-hold at the Ethos therapy system. Overall, 91 dose fractions, 4–15 per patient, were analysed. For the first 5 patients, treatment plans were optimized based on the goal list according to method 1, the second 5 according to goal list of method 2. 70.3% of the dose fractions were delivered using the adaptive plan, 29.6% using the scheduled plan. Main reasons for choosing the adaptive plan were better CTV coverage or improved dose homogeneity in 22 cases (24%), or improved organ-at-risk sparing in 36 cases (40%). In 6 cases (7%), the decision was based on visual preference. Patient, tumor, and treatment characteristics are given in Table 1.

**Table 1:** Patients, tumors and treatment characteristics

**Gender**	
Female	4
Male	6
**Age**	
Median (years)	73
Range (years)	48–82
**TNM-Stage classification**,** 8th edition**	
IIb	1
IIIa	5
IIIb	3
IIIC	1
**Primary tumor site**	
Upper lobe	4
Central	2
Lower lobe	4
**Performance status**	
ECOG 0	1
ECOG 1	8
ECOG 2	1
**COPD**	
Grade 0–1	6
Grade 2	4
**Response to induction treatment**	
CR	1
PR	6
NC	2
PD	1
**CTV volume (ccm)**	
Median	324.4
Range	98.3–653.9
**IMRT/ VMAT**	
9-Field	3
12-Field	6
VMAT	1
**Time from CBCT2 to end of treatment (**min)	
Median	7.7
Range	5.9–10.2

All numbers represent counts of patients according to the indicated characteristic category, except in the rows in which medians of continuous quantities are given. COPD Gold: chronic obstructive disease Global Initiative for Chronic Obstructive Lung Disease; CR, PR: complete or partial response according to Response evaluation criteria in solid tumours (Recist V1.1); NC-PD: no change or progressive disease; TN categories were defined according to the 8th edition of the TNM classification of the UICC.

**Fig. 1:**
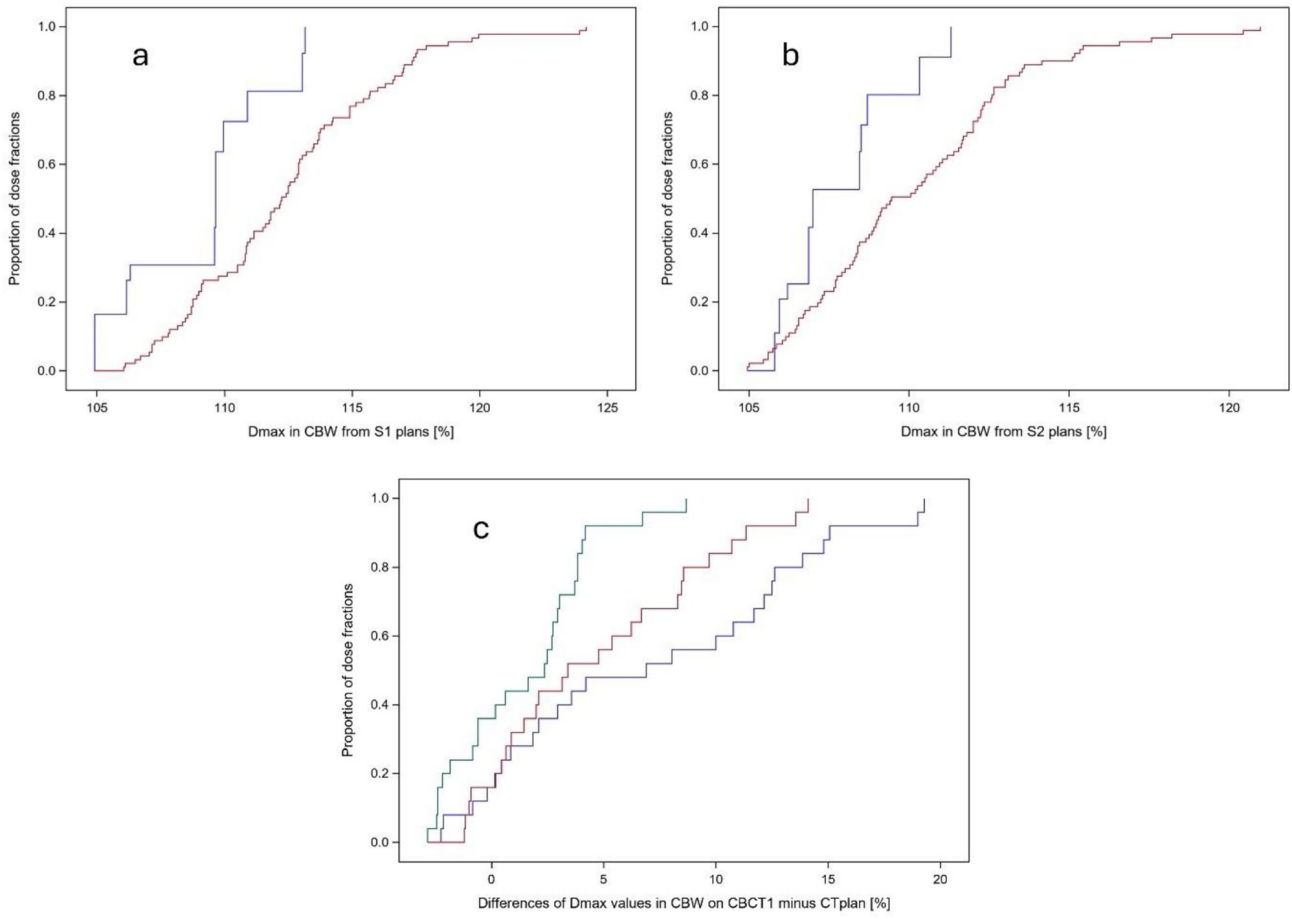
Empirical distribution functions of the Dmax values in the central bronchial wall (CBW) from the 10 scheduled plans of the 10 patients calculated according to method S1(**a**) and method S2 (**b**) on CTplan (blue step function) and on subsequent CBCT1 for the 91 dose fractions (red step function). Panel (**c**) shows the differences in CBW Dmax values between CBCT1 and CTplan, calculated using scheduled plans from method S1 (blue), method S2 (red), and method S3 (green). This analysis includes 25 dose fractions from the two patients whose plans were identified as least robust based on DeltaDmax (CBAL_PTV_)

The Dmax and D1cc values in the CBW obtained from the S1, S2, or Sclinical scheduled plans and the adaptive plans on CBCT1 and CBCT2 are given in Suppl Table 1a together with all other parameters analysed form the dose distributions on CBCT1 and CBCT2 from the 91 dose fractions as well as on the planning CT. In addition, a good agreement was found for the scheduled plans between the Ethos Acuros and Eclipse Acuros calculated dose distributions on CTplan (median g_2%2 mm_ value of 99.8% (range: 89 − 100%)). In the following, Eclipse Acuros dose distributions were analysed. Figure 1a shows the empirical distribution functions of the Dmax values in CBW from the scheduled plans for the 10 patients according to method 1 on CTplan and the 91 Dmax values on CBCT1. In a stratified comparison according to patient, the differences in Dmax values between CTplan and CBCT1 were significant (*p* < 0.0001, stratified Wilcoxon test). The median Dmax value in CBW from the different plans on CTplan, CBCT1, or CBCT2 are shown in Suppl. Table 1a. In addition, the D1cc values in CBW also showed significant but smaller differences between CTplan and CBCT1 (*p* < 0.0001, stratified Wilcoxon test). The maximal Dmax increase in CBW for the scheduled plans according to method 1 observed over the dose fractions per patient ranged from 0.85 to 19.3%, in median 3.8%. The maximum of D1cc increase in CBW ranged from − 2.7 to 7.3% per patient, median 1.9%.

Figure 1b shows the respective empirical distribution functions of the Dmax values in CBW according to method 2. Again, there were significant but smaller increase in Dmax between CTplan and CBCT1 detected (*p* < 0.0001, stratified Wilcoxon test). The D1cc values in CBW from method 2 scheduled plans also showed significant differences between CTplan and CBCT1 (*p* < 0.0001, stratified Wilcoxon test). In pairwise comparisons per dose fraction, the Dmax values in CBW on CBCT1 from the scheduled plans according to method 1 were moderately larger by a median value of 1.8% (range: -3.05 − 7.35%) than the respective Dmax values according to method 2 (*p* < 0.0001, signed rank test). Dmax values from the scheduled plans calculated with Acuros were compared with Dmax values from Monte Carlo calculation applied in ProSoma and a good agreement was found with a median difference of 0.65% (range: -1.8 − 1.9%) of the prescribed dose. Suppl Table 1b shows the correlation matrix and a hierarchical cluster diagram for the Dmax values in CBW on CBCT1 or CBCT2 from the different dose fractions and on the planning CT per patient for the S1, S2, Sclincal and Adapt plans. There was a high correlation of the Dmax values of the adaptive plans on CBCT1 and the subsequent CBCT2 with a correlation coefficient of 0.93. However, the correlation between Dmax values in CBW from the scheduled plans according to both method 1 and method 2 on CTplan and the subsequent CBCT1 was much smaller with correlation coefficients < 0.35.

Using dose accumulation, the Dmax and D1cc values of the accumulated scheduled plans according to method 1 in CBW had a median of 107.9% (range: 103.8–110.4%) and 105.9% (range: 102.5 − 107.1%) over the 10 patients. The respective Dmax and D1cc values in CBW of the accumulated S2 plans were 108.5% (range: 102.9 − 108.7%) and 103.3% (range: 101.1 − 106.9%). With respect to expected side effects on normal tissues in CBW, the accumulated values according to all plans were acceptable. Reasons for the lower Dmax values in CBW of the accumulated plans than the average value over the Dmax values from the different dose fractions are the spatial heterogeneity of the localisations of the Dmax values in CBW from dose fraction to dose fraction, so that the different Dmax values do not accumulate at the point. The overall mean deviation of the Dmax position from its mean position per patient from the scheduled plans according to method 1 was 16.3 mm without significant differences from patient to patient (*p* = 0.2596, Kruskal-Wallis-Test).

To further reduce plan sensitivity of Dmax in the CBW to anatomic variations in the subsequent CT, scheduled plans for two patients were recalculated according to method 3 by reducing the PTV overlap with CBW to 3 mm. The two patients with the largest DeltaDmax_CBAL_PTV__CTplan + WOR/CTplan were selected. The empirical distribution functions of the pairwise differences of the Dmax values in CBW on CBCT1 minus Dmax in CBW on CTplan per dose fraction are shown in Fig. 1c using scheduled plans according to method 1 (blue step function), method 2 (red step function), and method 3 (green step function) for the 25 dose fractions from these two patients. Plans optimized according to method 3 show the smallest Dmax deviations in CBW on CBCT1 in comparison to CTplan, followed by method 2 and method 1 (*p* < 0.0001, signed rank test for pairwise comparisons per dose fraction).

**Fig. 2:**
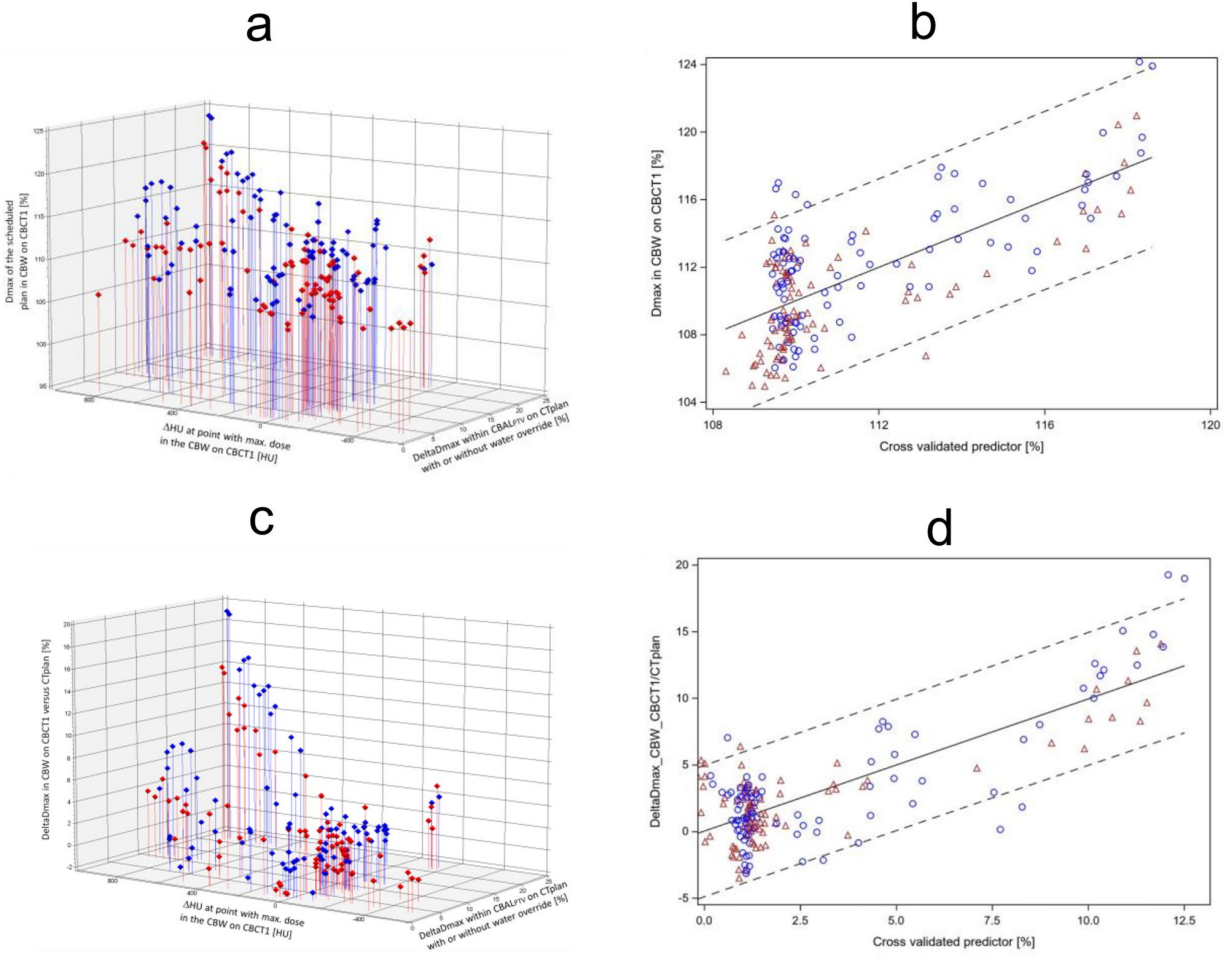
Dmax values in CBW by the scheduled plans on CBCT1 or DeltaDmax values within CBW calculated on CBCT1 and CTplan using the scheduled plans are depicted for the 91 dose fractions from the 10 patients. As independent characteristics, density changes at the Dmax point in the CBW on CBCT1 between CBCT1 and CTplan (ΔHU_CBCT2/CBCT_@DmaxP_CBW_CBCT1_S1/S2) and a parameter for plan sensitivity to density changes in the CBAL_PTV_ CTplan with or withut water override (DeltaDmax_CBAL_PTV__CTplan *±* WOR/CTplan_S1/S2) were used. Data from the scheduled plan according to method 1 are given with blue circles and according to method 2 with red triangles. **a**, Dmax values in CBW by the scheduled plans on CBCT1 are shown in dependence on the ΔHU_CBCT1/CTplan_@DmaxP_CBW_CBCT1_S1/S2 values from the same dose fraction and the plan sensitivity parameter DeltaDmax_CBAL_PTV__CTplan + WOR/CTplan_S1/S2. **b**, The dependence of the observed Dmax values in CBW on CBCT1 on the 5-fold cross validated predicted values according to the linear model build from the data shown in **a**. This model uses ΔHU_CBCT1/CTplan_@DmaxP_CBW_CBCT1_S1/S2 and DeltaDmax_CBAL_PTV__CTplan + WOR/CTplan_S1/S2 as main effects and a multiplicative interaction term of both factors to predict the Dmax values in CBW on CBCT1. **c**, Scatter plot of the DeltaDmax values within CBW calculated on CBCT1 and CTplan using the scheduled plans per dose fraction. **d**, 5-fold cross validated prediction of the DeltaDmax_CBW_CBCT1/CTplan_S1/S2 values. The linear model is built from the data shown in **c**

A linear model was built to predict *Dmax_CBW_CBCT1_S1/S2 on the subsequent CT*,* CBCT1*, a parameter for the sensitivity of the dose distribution from the plan to the anatomic changes (Fig. 2a). The independent parameters were *DeltaDmax_CBAL*_*PTV*_*_CTplan + WOR/CTplan_S1/S2* and ΔHU_CBCT1/CTplan_@DmaxP_ CBW_CBCT1_S1/2, parameters for dose deviations by density changes in the CBAL in the planning CT alone and density changes between CTplan and CBCT. The *DeltaDmax_CBAL*_*PTV*_*_CTplan + WOR/CTplan_S1/S2* of the used scheduled plans according to method 1 or 2 ranged from 1.7 − 22.1%, median 7.9%. The linear model showed that Dmax in the CBW on CBCT1 was significantly dependent on the multiplicative interaction effect of the two factors, DeltaDmax_CBAL_PTV__CTplan + WOR/CTplan_S1/S2 and ΔHU_CBCT1/CTplan_@DmaxP_CBW_CBCT1_S1/2 (*p* < 0.0001, t-test). However, each explanatory factor alone as a main effect was not significant (*p* > 0.05, t-test), indicating that one factor alone, density changes in the absence of plan sensitivity in the CBAL_PTV_, or plan sensitivity to density changes in the absence of density changes between CBCT1 and CTplan is not sufficient to cause a change in Dmax, and that a high plan sensitivity to density changes is a moderator for the effect of these density changes. For the whole data set of 182 data points from the scheduled plans according to method 1 and method 2, the least squares estimate for the interaction term was 0.000387% *±* 0.000080% increase in Dmax per one-unit HU density change times 1% increase in DeltaDmax_CBAL_PTV__CTplan + WOR/CTplan_S1/2. The intercept of the linear model was 109.35 *±* 0.38%. The internal validity of the above linear model was assessed by 5-fold cross validation. At each iteration of the cross-validation loop, the multiplicative interaction term of both explanatory factors became significant at *p* < 0.0005 (chi2 test). Figure 2b shows the dependence of the observed Dmax in CBW on the cross validated predicted value by the linear predictive model. The cross validated model explained R^2^ = 50.7% of the variance of the Dmax values on CBCT1 by the explanatory factors. The Root mean squared error between actual observed values and the cross validated predicted values was 2.65%.

Similar results were obtained for the other endpoint increase of Dmax values within CBW from CTplan to CBCT1 by applying scheduled plans according to method 1 or 2, DeltaDmax_CBW_CBCT1/CTplan_S1/2, Fig. 2c. The one independent factor in the linear model, DeltaDmax_CBAL_PTV__CTplan + WOR/CTplan_S1/2, did not became significant as main effect (*p* > 0.05), but the other, ΔHU_CBCT1/CTplan_@DmaxP_CBW_CBCT1_S1/2, did (*p* = 0.0003, t-test). The dominant factor again was the interaction effect between both factors (*p* < 0.0001, t-test). Using 5-fold cross validation, the multiplicative interaction term was highly significant at each of the cross-validation loops (*p* < 0.0001, t-test). The relation between DeltaDmax_CBW_CBCT1/CTplan_S1/S2 and the cross validated predictor from the linear model is shown in Fig. 2d. Root mean squared error between actual observed values and the cross validated predicted values was 2.49%. The model explained 64.2% of the variance of the DeltaDmax_CBW_CBCT1/CTplan-S1/S2 values. Beyond cross validation, we tested the generalizability of the models for Dmax and deltaDmax values with available data from another lung cancer patient from the prospective registry trial who was treated with adaptive VMAT radiotherapy at the Ethos therapy system equipped with a conventional CBCT before the upgrade with the Hypersight CBCT. Here, the synthetic CT scans before and after plan adaption were available from 10 dose fractions capturing the anatomic scenarios. The synthetic CT from the Ethos system is the planning CT deformed onto the conventional CBCT. The DeltaDmax_CBAL_PTV__CTplan + WOR/CTplan_S1 parameter for scheduled plan instability was 6.1%. The root mean squared error of the observed Dmax_CBW_CBCT1 values from those of the model was 2.87%, and those of DeltaDmax_CBW_CBCT1/CTplan_S1 was 1.89%, indicating a good prediction.

**Fig. 3:**
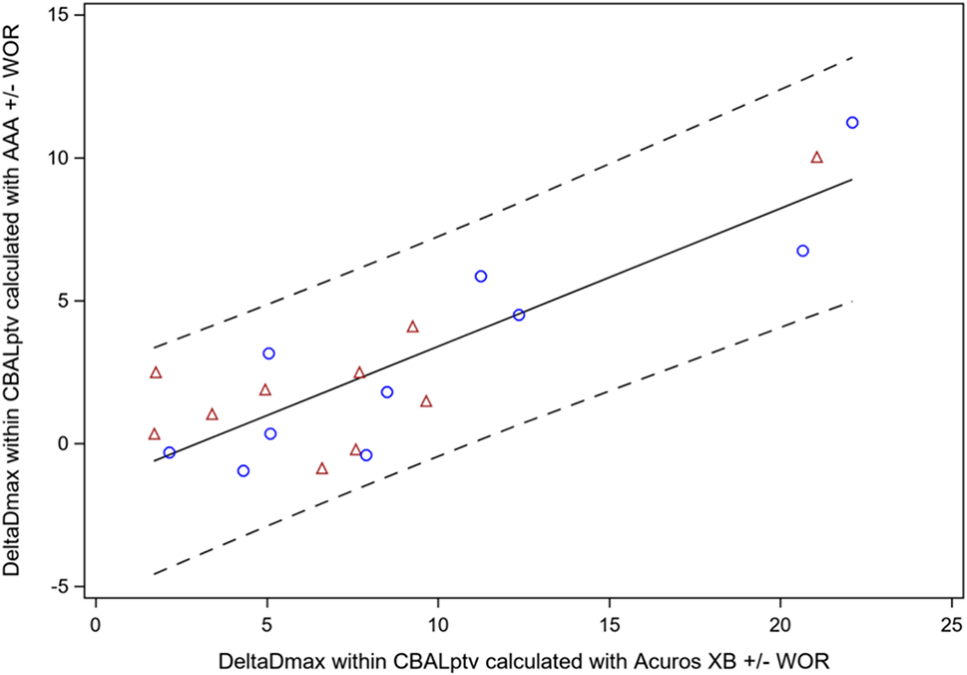
Relation between DeltaDmax values within CBAL_PTV_ on CTplan calculated using Acuros with or without water override versus the same parameter calculated with a AAA algorithm. Blue circles: scheduled plans according to method 1; red triangles: scheduled plans according to method 2

As AAA dose calculation algorithm shows less sensitivity of the dose distribution to density changes within air cavities, we compared the DeltaDmax_CBAL_PTV__CTplan + WOR/CTplan_S1/2 from Acuros XB dose calculation with those from AAA dose calculation. In fact, there was a close linear relation between both factors with a slope of 0.483 *±* 0.066 and a non-significant intercept of -1.435 *±* 0.696% (Fig. 3). The variance explained by the model was 74.8%. The best regression line through without an intercept had a slope of 0.372 +/- 0.041 demonstrating the markedly reduced sensitivity of dose distributions by the AAA algorithm to density changes in air cavities than the Acuros XB algorithm.

**Fig. 4:**
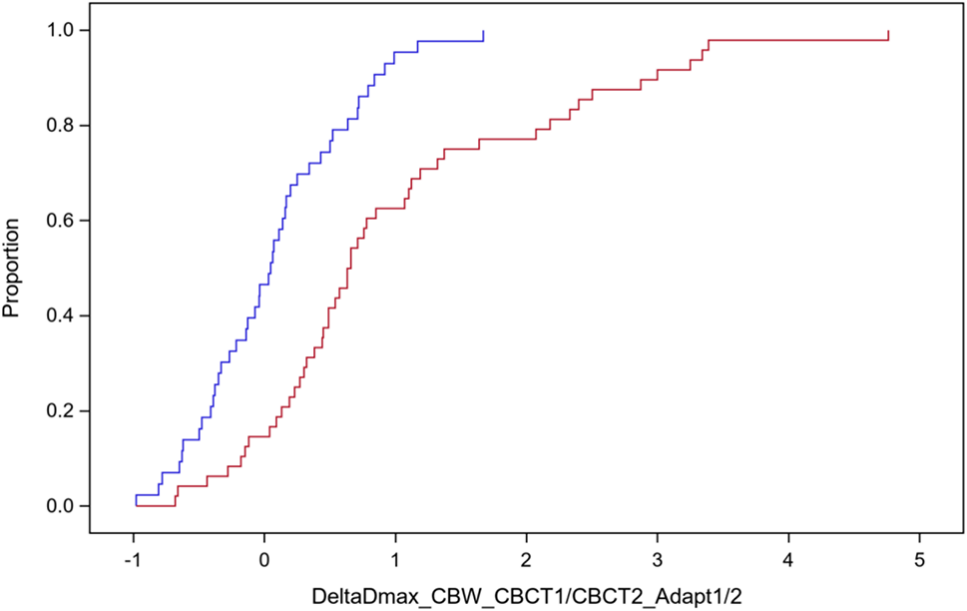
Empirical distribution functions of the DeltaDmax values in the central bronchial wall from adaptive plans calculated an CBCT1 and CBCT2. a, DeltaDmax values from 5 patients with 48 fractions treated with adaptive plans plans optimized according to method 1 (red step function). The red step function shows the DeltaDmax values from the 5 patients with 43 fractions and adaptive plans optimized according to method 2

In a next step, we analysed the sensitivity of the Dmax values in CBW using the adapted plans to anatomic changes between CBCT1 and the subsequent CBCT2. Figure 4 shows the empirical distribution functions of the DeltaDmax values in CBW from the 48 adapted plans calculated according to method 1 on CBCT1 or CBCT2 for 5 patients (blue step function). The respective distribution function for the 43 adapted plans according to method 2 from the 5 other patients is shown as a red step function. The distribution of the DeltaDmax values in CBW from the adaptive plans according to method 1 were significantly different from 0% (*p* < 0.0001, signed rank test) while the respective values from plans according to method 2 were not (*p* = 0.54, signed rank test). That indicates the higher robustness of plans optimized according to method 2 with respect to the effect of density changes in the CBAL on dose homogeneity.

**Fig. 5:**
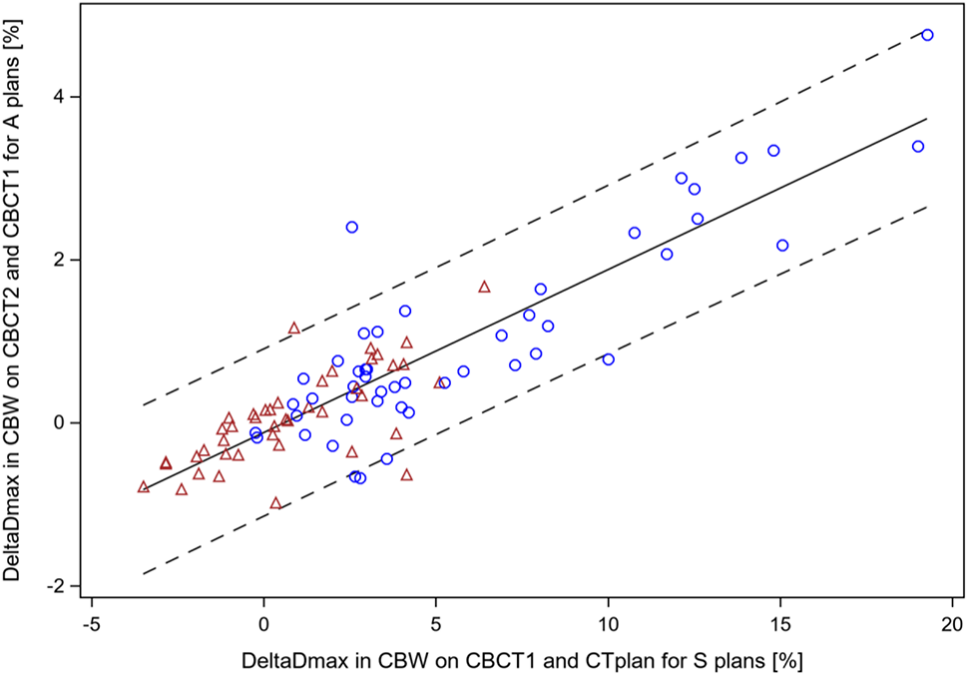
Relation between sensitivity of DeltaDmax in CBW from S (scheduled) and A (adapted) plans to anatomical changes in the subsequent CT of the 91 delivered dose fractions. Blue circles indicate data points from plans optimized according to the goal list of method 1 and the red triangles according to method 2. In pairwise comparisons of data from the same dose fraction, adapted plans showed smaller DeltaDmax values than the respective scheduled plans (*p* < 0.0001, signed rank test)

Comparing the adaptive plans with the corresponding scheduled plans according to the optimization method used in the clinic, the sensitivity of Dmax in CBW from the adaptive plans to anatomic changes on subsequent CT was markedly smaller than that of the respective scheduled plans (Fig. 5). The linear dependence of DeltaDmax in CBW between CBCT2 and CBCT1 using the adaptive plans on DeltaDmax in CBW between CBCT1 and CTplan using the scheduled plans showed a non-significant intercept of -0.121% + 0.067% and a slope of 0.200 +/- 0.011 (*p* < 0.0001, t-test). The Pearson correlation coefficient was 0.88 (95% CI: 0.82–0.92).

**Fig. 6:**
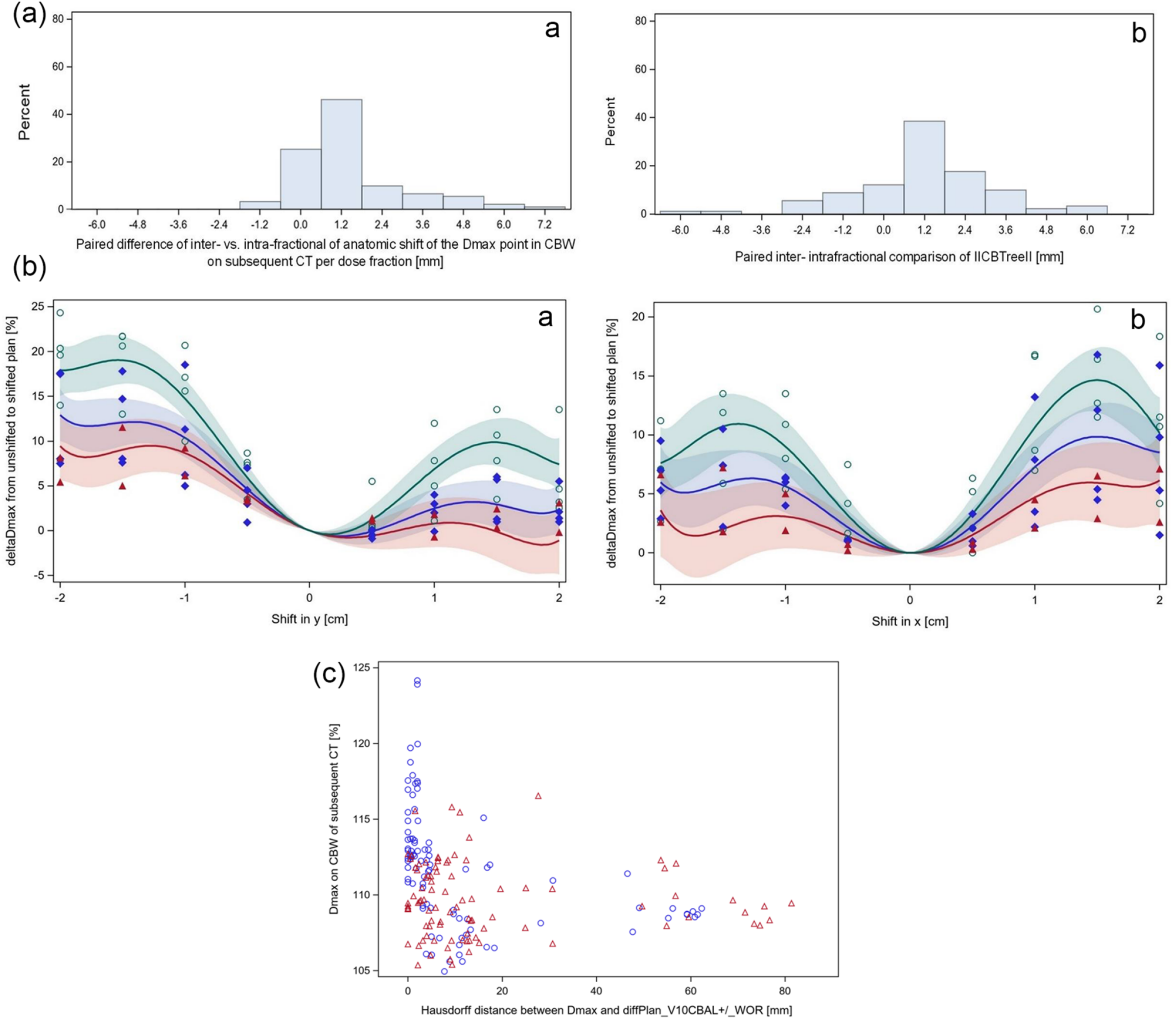
**a**: Distributions of paired comparisons of inter-versus intra-fractional anatomic deviation of the respective parameter from the same dose fraction over all 91 dose fractions. a, the first parameter is the length of the anatomic shift of the Dmax point in CBW of the subsequent CT as determined by elastic deformation from the subsequent to the source CT. The respective shift of Dmax in CBW on CBCT2 by the adaptive plan to CBCT1 was recorded as intra-fraction motion. b, the second parameter is||CBCTree||, the length of the rigid structure based coregistration vector of the central bronchial tree contoured on subsequent and source CT after online match. The medians of the inter-fractional deviations were larger than of the intra-fractional deviations for both parameters (*p* < 0.0001, signed rank test). **b**: *The isocenter of the scheduled plans according to method S1 (green curve*,* open green circles)*,* S2 (blue curve*,* closed blue diamonds)*,* and S3 (red curve*,* red closed triangles) was systematically shifted in±* *y (a) or±* *x (b) direction of the Dicom coordinate system in CTplan. Data were fitted by 6th degree polynomials. The 95% confidence bands for the expected predicted values were filled with the respective semi opaque colour. Dmax increases in CBW according to the length of the shift were largest for plans according to method S1*,* followed by S2*,* and S3.***c**: *Relation between Dmax on subsequent CT and the Hausdorff distance between the Dmax point in CBW on the subsequent CT backdeformed to the source CT by elastic deformation and the volume that shows sensitivity to water override in the trachea (V10*_*CBAL*_*from the difference plan in CBAL of the source CT with or without water override) Blue circles indicate data points from the clinically used scheduled plans*,* red triangles from the adaptive plans*

Next, we analysed the differences between intra- and inter-fractional anatomic deformations of CBW that could cause differences in the robustness of adaptive and scheduled plans with respect to dose homogeneity on the subsequent CT. Figure 6a shows the distribution of the paired differences between inter- and intra-fractional deformations of two parameters for the anatomic changes per dose fraction over the 91 dose fractions. The first parameter is the length of the anatomic shift of the Dmax point in CBW of the subsequent CT between source and subsequent CT by elastic deformation, Shift_source CT/subsequent CT_@DmaxP_CBW_subsequent CT_Planx. The second parameter was the length of the rigid 3D co-registration vector between CBW, contoured on the subsequent and the source CT,||CBCTree||. The medians of inter-fractional deviations were larger than those of the intra-fractional deviations for both measures (*p* < 0.0001, signed rank test, Fig. 6, SupplTable 1). The parameter from the anatomic deviations of the CBW that correlated best with *ΔHU*_*CBCT1/CTplan*_*@DmaxP_CBW_CBCT_Sclinical* was the absolute value of the y component of the deformation vector at the Dmax point in CBW of the subsequent CT (Persons’s correlation coefficient *r* = 0.43 (95% CI: 0.25–0.58). For comparison with other studies, the inter-fractional systematic error of||CBCTree|| in the x, y and z Dicom coordinate direction was 1.32 mm, 1.59 mm, and 2.23 mm, with a respective inter-fractional random error of 0.88 mm, 1.15 mm, and 0.93 mm. The intra-fractional systematic error was 0.18 mm, 0.91 mm and 0.79 mm in x, y and z and the inter-fractional random error was 0.68 mm, 1.35 mm, and 1.11 mm. The resulting PTV margins were 3.9 mm, 4.8 mm, and 6.2 mm for inter-fractional and 0.9 mm, 3.2 mm, and 2,8 mm for intra-fractional deviations in x, y or z direction respectively, according to the van Herk formula [45].

Systematic shifts of the isocenters of the scheduled plans according to methods S1 and S2 on CTplan were performed in *±* x and *±* y direction for the 4 patients with largest plan sensitivity on CTplan for density changes in the CBALPTV, *DeltaDmax_CBAL*_*PTV*_*_CTplan + WOR/CTplan*, all with values > 11%. Plans were also optimized according to method 3 for two of these patients. The Dmax values in CBW after shifts were determined and compared to the Dmax values of the respective unshifted CTplan. Figure 6b shows a 6th degree polynomial fit to the data. The increase in Dmax from shifts in x or y direction up to +/- 1.5 cm was most pronounced for plans according to method S1 and highly significant (*p* < 0.0001, t-test). In comparison to S1, S2 and S3 optimized plans showed smaller increases according to the quadratic term (*p* = 0.0003 or *p* < 0.0001, respectively, t-test). Shifts larger than 1.5 cm did not further increase the Dmax values in CBW. There were no significant inter-patient differences of the effects of systematic shifts on Dmax in CBW.

The Hausdorff distance of the Dmax point in CBW on subsequent CT back-deformed to the source CT from the volume that shows sensitivity to water override within the central bronchial tree on the source CT (*V10*_*CBAL*_) is given in Fig. 6c on the x-achsis. The scatter plot shows Dmax in CBW in dependence on the Hausdorff distances HD(diffplanV10_CBAL_, DmaxP_CBW_CBCT1)_CTplan for the scheduled plans clinically used (blue circles) and the adaptive plans (red triangles). There was a significantly negative correlation with a Spearman correlation coefficient of -0.50 (95% CI: -0.60 - -0.38). Dmax above 117% were only observed for small Hausdorff distances using the scheduled plans. This points to a causal relation of Dmax and the increase photon fluence in CBAL in Acuros optimized plans.

Finally, we analysed the EUD values in the central CTV (EUD_cCTV_) by the scheduled plans on CTplan and CBCT1 and of the adaptive plans on CBCT1 and CBCT2. The EUD_cCTV_ of the clinical scheduled plans according to method 1 on CTplan had a median value of 101.1% (range 99.7 − 102.0%) and on CBCT1 of 101.2% (range: 94.5 − 102.9%), with marginal differences between CTplan and CBCT1 (*p* = 0.0481, Wilcoxon test, stratified by patient). The clinical scheduled plans according to method 2 showed slightly larger decreases of the EUD_cCTV_ on CBCT1 in comparison to CTplan (*p* = 0.0023, stratified Wilcoxon Test). The median EUD values on CTplan were 101.6% (100.9 − 102.0%) and on CBCT1 100.6% (range: 90.4 − 103.4%). However, dose accumulation over all dose fractions demonstrated that the EUD in the central CTV stayed > 95% for all patients on CBCT1 with scheduled plans according to method 1 and 2. The same holds for the adaptive plans. The EUD_cCTV_ values from the the adaptive plans had a median value of 101.5% (range: 98.1% − 103,6%) on CBCT1 and of 101.3% (97.5 − 103.3%) on CBCT2.

## Discussion

In stereotactic radiotherapy of peripheral lung tumors, AAA algorithms, as improved pencile beam algorithms, overestimate the D98% or D95% in the PTV. On average, the differences range from 1.8 to 3.2% but up to 12% in some patients in comparison to the Acuros XB algorithms reporting dose to medium due to underestimation of the build-up effects near the tumor-lung interfaces [46–51]. Analysing a large patient registry of 928 patients treated with stereotactic radiotherapy for stage I non-small cell lung cancer, Ohri et al. found that patients using treatment plans optimized on pencil beam instead of Monte Carlo dose calculations have an increased risk of local recurrence using multivariable proportional hazards analysis with inclusion of additional parameters as biologically effective dose and tumor diameter [52]. Other studies compared Acuros and AAA plans for head and neck cancer patient with air cavities within or around the planning target volume. In general, AAA plans overestimated the tumor control probabilities or the D95% values for the PTV when compared to Acuros by on average 1.3 − 2.6% [18, 53, 54]. In comparison to the Acuros algorithm, the dose in the air cavities is overestimated and the dose in bone underestimated by the AAA algorithms [23, 54, 55]. This effect will influence the dose-volume histograms for the PTV if it overlaps these structures. A head and neck phantom study by Ito et al. found that if the air content within the PTV increases, the D95% for the PTV of the same delivered treatment plan calculated with AAA increased in comparison to calculation with Acuros [56]. Similarly, in prostate bed radiotherapy with an endorectal balloon, the AAA algorithm estimated higher D95% values for the PTV overlapping with the air-filled balloon than those calculated with Acuros [57].

In contrast to the above studies that analysed AAA optimized plans, treatment plans of the present study were optimized based on Acuros dose calculation and the effect of density changes due to inter- and intra-fractional anatomic deformations was analysed. Larger inter-fractional dose deviations of the scheduled plans were found in the CBW > 7% in 37% of the delivered dose fractions. A linear model identified the interaction effect between the Dmax difference within CBAL_PTV_ with or without water override and the Hounsfield Unit change from source to subsequent CT at the Dmax point in the subsequent CT as predictive for the Dmax value in CBW of the consecutive CT as the most predictive factor. This can be interpreted that the Dmax difference in CBAL_PTV_ with or without water override in the source CT is a moderator of the effect of density changes between source and subsequent CT on Dmax deviations in CBW [58]. In the present study, it is demonstrated that plan sensitivity to water override in the CBAL between scheduled and adaptive plans are highly correlated and therefore can be controlled during the initial planning steps by the generation of the scheduled plans. Reducing the PTV overlap with CBAL and relaxing the goals for PTV coverage in relation to CTV coverage can mitigate plan sensitivity to anatomic changes with respect to dose homogeneity in CBW. Intra-fraction motion of the bronchial tree from breath hold to breath hold is the cause of intra-fractional density changes at the Dmax location of the dose distribution in the CBW. In the present study, PTV margins between 0.9 mm and 3.2 mm were found necessary for intra-fractional anatomic deviations in latero-lateral and anterior-posterior direction. These intra-fractional deviations were similar to those in the studies on surface guided radiotherapy of breast cancer or stereotactic body radiotherapy of lung cancer in deep inspiration breath hold [26, 59] and were substantially smaller than inter-fractional deviations. Analyses of systematic translational shifts of the iso-center within the patient showed, that deviations larger than 7 mm in x or y were necessary to increase the dose within the CBW substantially by > 7% and therefore, the intra-fractional deviations, but not the inter-fractional deviations, were small enough to avoid these dose-increases. In principle, image guidance according to the central bronchial tree could minimize inter-fractional deviations in the CBW, but this will have the consequence of larger necessary PTV margins around a peripheral primary tumor than considering also the position of the primary tumor for image guidance [60].

Acuros dose calculation algorithms was extensively compared with Monte Carlo algorithms with respect to low density cavities in other studies and was found to have overall comparable accuracy [21–23, 55, 61]. Dmax within the CBW was compared in this study between Prosoma MC and Acuros and a good agreement of the Dmax values in CBW was found within 2% of the prescribed dose. Dose within the air cavity CBAL_PTV_ by Acuros XB in the planning CT with or without WOR was evaluated as predictive factor in this study and found it as a moderator for the Dmax in CBW of the subsequent CT.

Weaknesses of this study were, that VMAT plans were underrepresented with only one patient received a VMAT plan. However, dose optimization during the adaptive workflow is much more time consuming for VMAT than for IMRT plans, in this study on average by about 7 min, so that IMRT planning was the preferred treatment option. Although the variability of the planning target volumes with respect to the central bronchial tree were considerable, the number of scheduled plans with more instable plans characterized by DeltaDmax_CBAL_PTV__CTplan + WOR/CTplan values > 15% is moderate so that the precision of the prediction of the dosimetric effect of anatomic changes on Dmax in CBW of this study is limited. However, the precision was found adequate as tested by an additional treatment series with a VMAT plan from our prospective registry out of the trainings data for the present study.

No phantom measurements were performed, however those studies were extensively performed in depth [16, 18, 22, 38]. Instead, detailed analyses the clinical data set was performed with the Acuros algorithm on the available Hypersight™ CBCT.

In conclusions, dose homogeneity in CBW of treatment plans optimized on the basis of Acuros dose calculation in the Ethos therapy system was found to be sufficiently robust against intra-fractional deformations during course of online adaptive radiotherapy irradiating in deep inspiratory breath hold. Plan sensitivity to larger interfractional anatomic changes was found larger but can be detected on the planning CT by the WOR of air in CBAL_PTV_. With decreasing overlap of the PTV with the central bronchial tree, the robustness of the Ethos plans against anatomic changes with regard to dose homogeneity in CBW increased.

## Electronic supplementary material

Below is the link to the electronic supplementary material.

Supplementary Material 1

Supplementary Material 2

## Data Availability

No datasets were generated or analysed during the current study.
