# Supplementary material for: Are treatment plans optimized on the basis of acuros XB dose calculation robust against anatomic changes during online adaptive radiotherapy for lung cancer regarding dose homogeneity?
Source: Radiat Oncol. 2025 May 15;20:75. doi: 10.1186/s13014-025-02656-1 (PMC12082940; doi:10.1186/s13014-025-02656-1)
Supplement: Supplementary file 1 — Supplementary Material 1 [file 13014_2025_2656_MOESM1_ESM.pdf]

West German Tumour Centre

Radiotherapy clinic Director: Prof. Dr med. M. Stuschke

		Phone +49 (0) 201 723
	2320 Station S1	Phone +49 (0) 201 723
	2041 Station S2	Phone +49 (0) 201 723
	2042	Fax +49 (0) 201 723 5960
Director of Studies:	Prof. Dr med. M. Stuschke	
Deputy:	Prof Dr med C. Pöttgen	

PATIENT INFORMATION ON THE CLINICAL REGISTER

Dear patient,
We would like to ask you whether you are willing to take part in the registry study described below.

In the clinical registry, it is planned to scientifically evaluate the effects of radiotherapy in the long term, both in terms of effectiveness and the possible spectrum of side effects.
This study was reviewed and approved by the ethics committee of Essen University Hospital.

1. background and objectives of the register

The examinations you have undergone so far have revealed the presence of a malignant disease:

□ Malignant disease of the central nervous system

____

□ Malignant disease of the ENT / maxillofacial region

____ □

Malignant disease of the respiratory tract / lungs

____

ID: 149261 | ExD printed from roXtra on: 13.03.2025

□ Malignant disease of the digestive tract

____

□ Malignant disease of the urogenital tract

____

□ Malignant disease of the muscular/connective tissue/skeletal system

____

According to the recommendations of the interdisciplinary tumour conference or the
Guidelines the scientific Specialised societies following, is one radiotherapy to treat the disease.

The scientific recommendations for radiotherapy leave room for the treating team of doctors to decide on the specific procedure, so that the appropriate radiotherapy is carried out taking into account the variant that is considered to be the best possible treatment modality in each case.

Data collected in the course of clinical routine (tumour regression, freedom from disease, tolerability, side effect profile) will be used in the longer term for evaluations in various scientific projects.

This includes, for example, the comparison of mucosal side effects of twice-daily (accelerated) radiotherapy of lung carcinoma in relation to once-daily (conventionally fractionated) radiotherapy, or the comparison of the effectiveness and side effect profile of patients with prostate carcinoma treated by brachytherapy in relation to patients receiving percutaneous radiotherapy alone.

In addition, we would like to ask you if you are willing to provide us with a blood tube taken during routine clinical blood sampling for a limited number of additional analyses. These tests are carried out in the laboratory of the radiotherapy clinic or in a

The research will be carried out in the laboratory of the University Hospital and will be linked to your medical data in order to improve the detection and treatment of diseases. The aim of this research is not to diagnose you or other individual Persons one Diagnosis to create or to prove disease-causing predispositions. Rather, the comparative examination of larger groups of people is intended to determine biomedical correlations. The samples are destroyed after testing and are not stored permanently.

ID: 149261 | ExD printed from roXtra on: 13.03.2025

For diagnostic or therapeutic reasons, blood samples will be taken during your treatment anyway. We would like to take up to 20 ml of additional blood.
This collection is not associated with any additional health risks for you. There are no health risks for you when using residual materials that are produced as part of your diagnostics/therapy.

2. voluntary participation and cancellation

Your participation in the register is voluntary. You can revoke your consent to participate at any time without giving reasons and without any disadvantages for you. Please send your cancellation to the contact address below.
If you withdraw your consent, the possibility of accessing your data in the context of the Clinical Register will be withdrawn.

3. data protection

The data documented by you as part of the study is collected pseudonymised and is subject to data protection.
Pseudonymised means that no names or initials are used, only a number and/or letter code, possibly with the year of birth. An assignment to your person is only possible via the pseudonymisation list, which is kept under lock and key by the study director.
Before data is released for specific scientific projects, a favourable vote is obtained from the Ethics Committee.
The data collected in this test will be analysed pseudonymously for scientific purposes and, if necessary, published anonymously. For this purpose, your personal data will be stored without mentioning your name. You can obtain information about which of your data has been recorded at any time.

In accordance with the European General Data Protection Regulation (EU GDPR), you have the right to:

- Information about the processing of your data,
- Correction or deletion of your data,
- Restriction of processing (only storage possible),
- Objection to the processing,
- Data portability,
- Revocation of your given consent with effect for the future,

ID: 149261 | ExD printed from roXtra on: 13.03.2025

- Complaint to the data protection supervisory authority.

The Radiotherapy Clinic is responsible for data processing within this study. The data collected as part of the clinical routine is recorded and analysed here and archived for 30 years for reasons of radiation protection law.
You have the right to receive information about your personal data and to have it corrected in the event of an error.
The evaluation of the data remains limited to the University Hospital; there are no plans to exchange data with external clinics.
The data protection concept was reviewed and approved by the local data protection officer.
Further details on the use of your data, security precautions, maintaining the confidentiality of your data and how to obtain copies can be obtained from the following person:

4. contact for questions, cancellation, etc.

Director of Studies

Prof. Dr Martin Stuschke, Prof. Dr Christoph Pöttgen, Clinic and Polyclinic for Radiotherapy,
Medical Faculty, University of Duisburg-Essen,
Hufelandstrasse 55,
45122 Essen, Germany
Tel +49 201 723 2322
Fax
+49 201 723 5960 Email: martin.stuschke@uk-essen.de; christoph.poettgen@uk-essen.de

The data protection officers at Essen University Hospital are also available to answer your questions:
Data Protection Officer
Essen University Hospital
Robert-Koch-Str. 9 - 11, 45147 Essen
Phone: 0201-723-1898 or -5179
E-mail: datenschutz@uk-essen.de

ID: 149261 | ExD printed from roXtra on: 13.03.2025

You also have the right to lodge a complaint with the competent supervisory authority:

State Commissioner for Data Protection and Freedom of Information North Rhine-Westphalia P.O. Box 20 04 44, 40102 Düsseldorf
Tel.: 0211-38424-0 Fax: 0211-38424-10
E-mail: poststelle@ldi.nrw.de

West German Tumour Centre

Radiological clinic

Director: Prof. Dr med. M. Stuschke

Phone +49 (0) 201 723 2320

Fax +49 (0) 201 723 5960

Declaration of consent Clinical Register

Name of the patient in block capitals

Born on

I was informed in writing and verbally about the above-mentioned study and all my questions were answered.

I am taking part in this study voluntarily.

I know that I can revoke my consent to participate at any time without giving reasons and without any disadvantages.

I agree that data collected as part of the study may be recorded on questionnaires and / or electronic data carriers and analysed without mentioning my name (pseudonymised). I also agree that the study data may be used in anonymised form for scientific presentations and publications.

I have received a copy of the information leaflet and the signed declaration of consent.

Name of the patient in block letters aben

Place, date

Signature of the patient

I have informed the patient about the nature, significance and scope of participation in the clinical register.
He/she has been given a copy of the patient information and consent form.

Name of the doctor in block capitals

sprinted from ro Xtra on: 13.03.2025

Place, date Signature of investigator

ID: 149261 | ExDau
