# Supplementary material for: Are treatment plans optimized on the basis of acuros XB dose calculation robust against anatomic changes during online adaptive radiotherapy for lung cancer regarding dose homogeneity?
Source: Radiat Oncol. 2025 May 15;20:75. doi: 10.1186/s13014-025-02656-1 (PMC12082940; doi:10.1186/s13014-025-02656-1)
Supplement: Supplementary file 2 — Supplementary Material 2 [file 13014_2025_2656_MOESM2_ESM.docx]

Suppl Table 1a: Parameters obtained from scheduled Plans on CTplan and CBCT1 and of adapted plans on CBCT1 to CBCT2.

| parameter | Median [range] |
| --- | --- |
| Dmax_CBW_CTplan_S1 [%] | 109.65 (104.90 – 113.15) |
| Dmax_CBW_CTplan_S2 [%] | 107.00 [105.80 – 111.30) |
| Dmax _CBW_CTplan_S_clincial_ [%] | 108.70 (104.90 – 111.30) |
| Dmax_CBW_CBCT1_S1 [%] | 112.50 (106.05 – 124.17) |
| Dmax_CBW_CBCT1_S2 [%] | 109.45 (104.95 – 120.97) |
| Dmax_CBW_CBCT1_S_clincial_ [%] | 111.80 (104.95 – 124.17) |
| deltaDmax_CBW_CBCT1/CBCT2_Adapt1/2 [%] | 0.34 (-0.98 – 4.76) |
| D1cc_CBW_CTplan_S1 [%] | 106.00 (102.70 – 107.85) |
| D1cc_CBW_CTplan_S2 [%] | 103.80 (101.70 – 108.35) |
| D1cc_CBW_CTplan_S_clinical_ [%] | 105.04 (102.70 – 108.35) |
| D1cc_CBW_CBCT1_S1 [%] | 107.60 (103.50 – 110.55) |
| D1cc_CBW_CBCT1_S2 [%] | 105.10 (101.95 – 109.20) |
| D1cc_CBW_CBCT1_S_clinical_ [%] | 106.60 (101.95 – 110.55) |
| Dmax_CBAL_PTV__CTplan_S1 [%] | 110.15 (105.60 – 113.05) |
| Dmax_CBAL_PTV__CTplan+WOR_S1 [%] | 117.95 (110.20 – 133.70) |
| Dmax_CBAL_PTV__CTplan_S2 [%] | 104.20 (99.95 – 110.60) |
| Dmax_CBAL_PTV__CTplan+WOR_S2 [%] | 110.95 (103.35 – 125.27) |
| Dmax_CBAL_PTV__CTplan_S_clinical_ [%] | 106.50 (104.05 – 110.60) |
| Dmax_CBAL_PTV__CTplan+WOR_S_clinical_ [%] | 115.85 (105.80 – 128.80) |
| Dmax_CBAL_PTV__CTplan_S_clinical_-AAA [%] | 108.90 (104.95 – 117.93) |
| ΔHU_CBCT1/CTplan_@DmaxP_CBW_CBCT1_S1 [HU] | 70.4 (-164.5 – 936.6) |
| ΔHU_CBCT1/CTplan_@DmaxP_CBW_CBCT1_S2 [HU] | -5.74 (-644.8 – 935.5) |
| ΔHU_CBCT2/CBCT_@DmaxP_CBW_CBCT2_Adapt [HU] | -1.91 (-287.8 – 720.0) |
| Shift_CTplan/CBCT1_@DmaxP_CBW_CBCT1_S_clinical_ [mm] | 1.7 (0-3 – 8.7) |
| Shift_CBCT2/CBCT1_@DmaxP_CBW_CBCT2_Adpat [mm] | 0.7 [0.0 – 2.3) |
| Shift_CBCT1/CTplan__\|\|CBTree\|\| [mm] | 2.9 (0.4 – 7.9) |
| Shift_CBCT2/T1__\|\|CBTree\|\| [mm] | 1.4 (0.3 – 8.6) |
| HD(diffplanV10_CBAL_, DmaxP_CBW_CBCT1)_  CTplan_Sclinical [mm] | 3.4 (0 .0 – 62.3) |
| HD(diffPlanV10_CBAL_, DmaxP_CBW_CBCT2)_  CBCT1_Adapt [mm] | 8.5 (0.0 – 81.3) |

Suppl Table 1a. **Dmax_CBW_CTx_Planx**: Dmax values in the CBW on CTx (CTplan, CBCT1, CBCT2) from PlanX (scheduled plans according to method S1, S2 or as clinically used (S_clinical_)). For the adaptive plans, deltaDmax_CBW_CBCT1/CBCT2_Adpat is given.; **D1cc_CBW_CTx_Planx:** D1cc_CBW is the minimum dose in the 1 cubic centimter of the CBW containing the voxel with the highest doses; **Dmax_CBAL_PTV__CTplan(+WOR)_Planx**: Dmax in the central bronchial air lumen overlapping the PTV in the planning CT using Planx (scheduled Plan according to the indicated method (S1, S2, S_clinical_)), + WOR indicates water override of the CBAL for dose calculation, AAA indicates the dose calcultion with the AAA algorithm; **∆HU_subsequent CT/source CT_@DmaxP_CBW_subsequnet CT_Planx:** The difference between the HU value at the Dmax position in CBW on the subsequent CT and the HU value at the same point on the sourceCT coergisterd by the clinical match; **Shift_source CT/subsequent CT_@DmaxP_CBW_subsequent CT_Planx:** Length of the deformation vector from the Dmax point in CBW on the subsequent CT by Planx to the source CT after hybrid deformation in MIM Maestro. The deformation vector was sampled over a sphere of 1mm radius, that was shifted by the deformation field; **Shift_source CT/subsequent_ CT_||CBTree||:** The length of the rigist registration vector between CBT contoured on sourceCT and subsequentCT after image fusion by the clinical match; **HD(diffplanV10_CBAL,_ DmaxP_CBW_subsequent CT)_Source CT_Planx:** the Hausdorff distance between diffplanV10CBAL as isodose-contour and the Dmax point in CBW on subsequent CT copied to the source CT. The diffplanV10CBAL is the volume in CBAL with a dose increase by more than 10% of the prescribed dose obtained from the dose difference-distribution between a Planx on the source CT calculated with or without water override of the CBAL.

Suppl Table 1b Correlation between Dmax values on CBW

|  | Dmax_CBW_ CBCT1_S2 | Dmax_CBW_ CBCT1_Streat | Dmax_CBW_ CBCT1_S1 | Dmax_CBW_ CBCT2_Adapt | Dmax_CBW_ CBCT1_Adapt | Dmax_CBW_ CTplan_S2 | Dmax _CBW_ CTplan_Streat | Dmax_CBW_ CTplan_S1 |
| --- | --- | --- | --- | --- | --- | --- | --- | --- |
| Dmax_CBW_ CBCT1_S2 | _1.0000_ | _0.8158_  _<0.0001_ | _0.7983_  _<0.0001_ | _-0.0844_  _0.4261_ | _0.2182_  _0.0378_ | _0.2347_  _0.0251_ | _-0.2084_  _0.0475_ | _-0.1132_  _0.2854_ |
| Dmax_CBW_ CBCT1_Streat |  | _1.0000_ | _0.9260_  _<0.0001_ | _-0.0248_  _0.8156_ | _0.3159_  _0.0023_ | _-0.1176_  _0.2669_ | _-0.1411_  _0.1821_ | _-0.1941_  _0.0652_ |
| Dmax_CBW_ CBCT1_S1 |  |  | _1.0000_ | _-0.0017_  _0.9876_ | _0.3153_  _0.0023_ | _-0.0048_  _0.9638_ | _-0.0825_  _0.4369_ | _-0.0159_  _0.8812_ |
| Dmax_CBW_ CBCT2_Adapt |  |  |  | _1.0000_ | _0.9343_  _<0.0001_ | _0.3567_  _0.0005_ | _0.5194_  _<0.0001_ | _0.5137_  _<0.0001_ |
| Dmax_CBW_ CBCT1_Adapt |  |  |  |  | _1.0000_ | _0.2941_  _0.0047_ | _0.3965_  _<0.0001_ | _0.3936_  _0.0001_ |
| Dmax_CBW_ CTplan_S2 |  |  |  |  |  | _1.0000_ | _0.6151_  _<0.0001_ | _0.6687_  _<0.0001_ |
| Dmax _CBW_ CTplan_Streat |  |  |  |  |  |  | _1.0000_ | _0.8613_  _<0.0001_ |
| Dmax_CBW_ CTplan_S1 |  |  |  |  |  |  |  | _1.0000_ |

Cross correlation table between the D **Dmax_CBW_CTx_Planx** values. There was a high correlation between the Dmax values of the adaptive plans on CBCT1 and the subsequent CBCT2 with a correlation coefficient of 0.93. However, the correlation between Dmax values in CBW from the scheduled plans according to both method 1 and method 2 on CTplan and the subsequent CBCT1 was much smaller with correlation coefficients < 0.35.


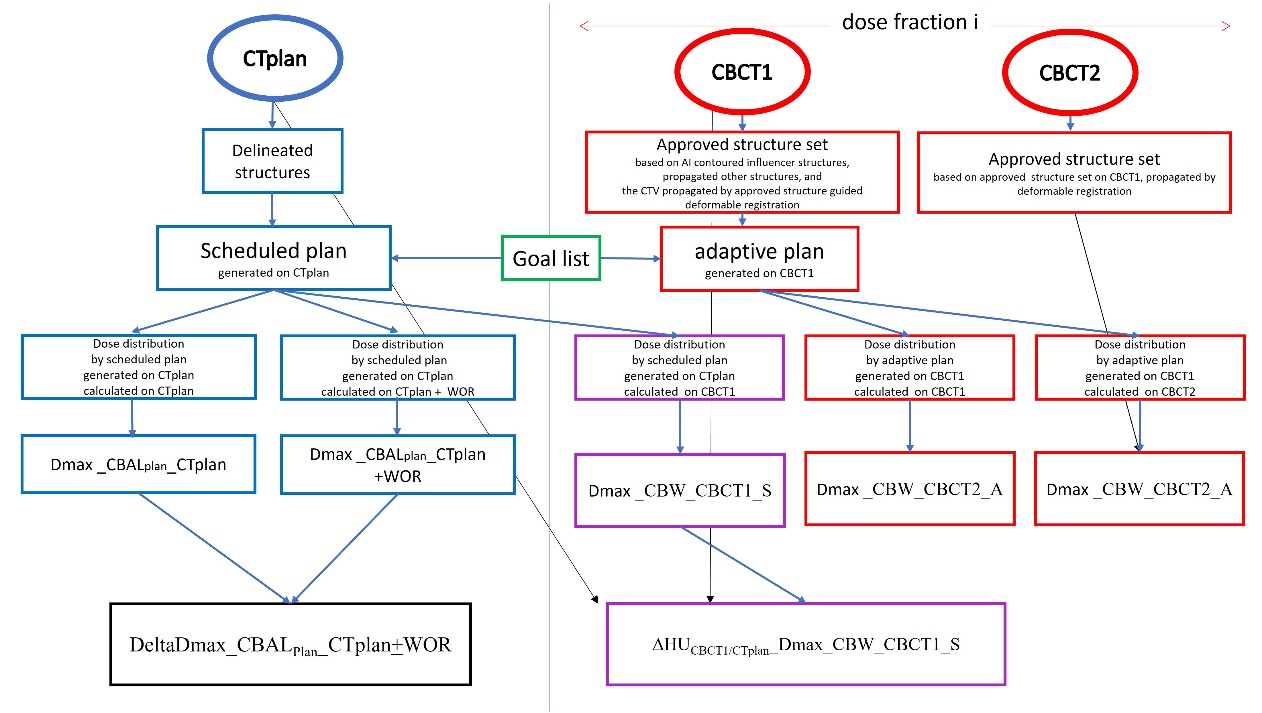


Suppl Figure 1: Flowchart illustration of the workflow used for the generation of the analysed parameters used for the predictive models of this study. Approved structure set implies, that all structures were supervised and changed if neccessary by the treating radiation oncologist.


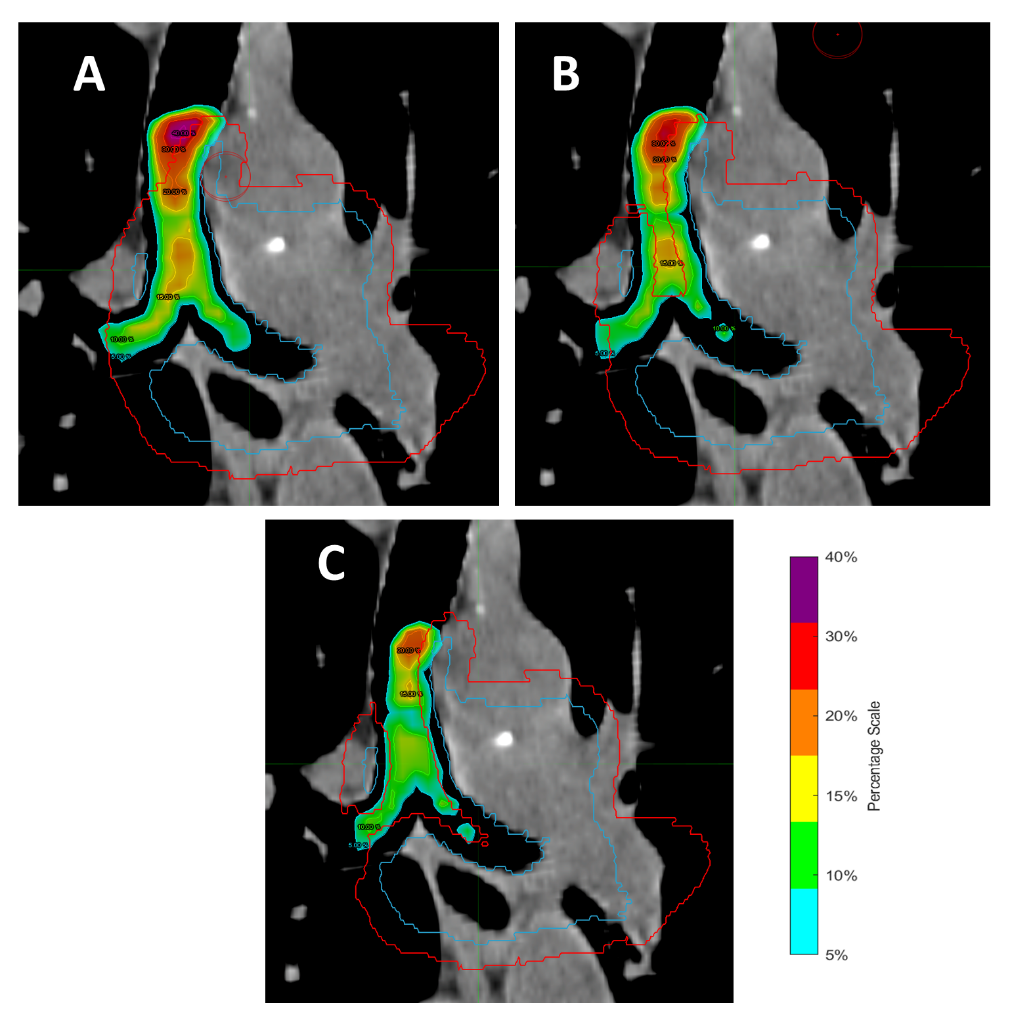


Suppl Figure 2: Dose difference distribution of the scheduled plans according to method S1 (a), S2 (b), and S3 (c) calculated with Acuros in Eclipse on CTplan with or without water override. Dose differences are given in percent of the prescribed dose. Blue lines indicate the boundaries of the CTV, red lines those of the PTV. Note, that the PTV differ between a, b, and c.


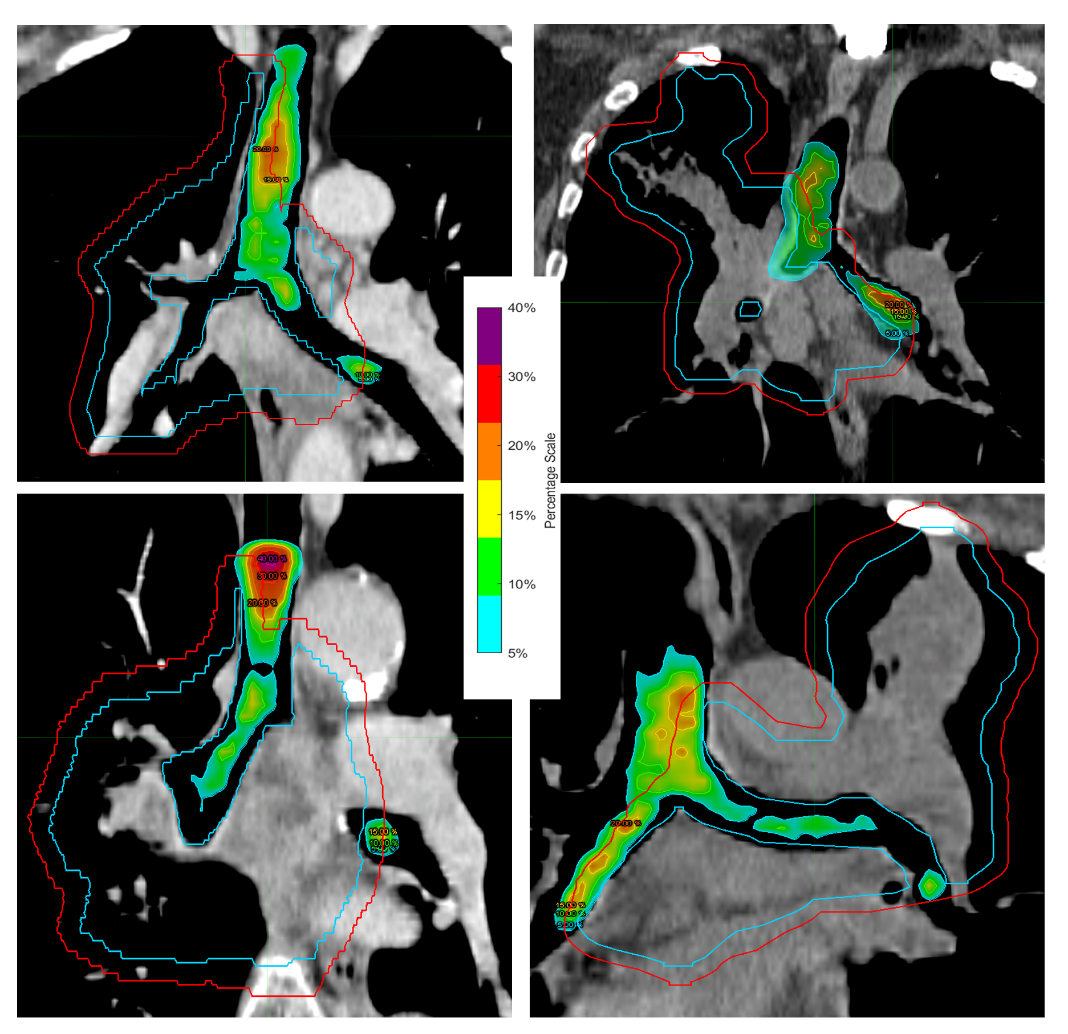


Suppl Figure 3: Dose difference distribution of the scheduled plans according to method S1 for four different patients, calculated with Acuros in Eclipse on CTplan with or without water override. Dose differences are given in percent of the prescribed dose. Blue lines indicate the boundaries of the CTV, red lines those of the PTV. The largest dose differences were found within the central bronchial air lumen (CBAL) where the PTV cross the CBAL.


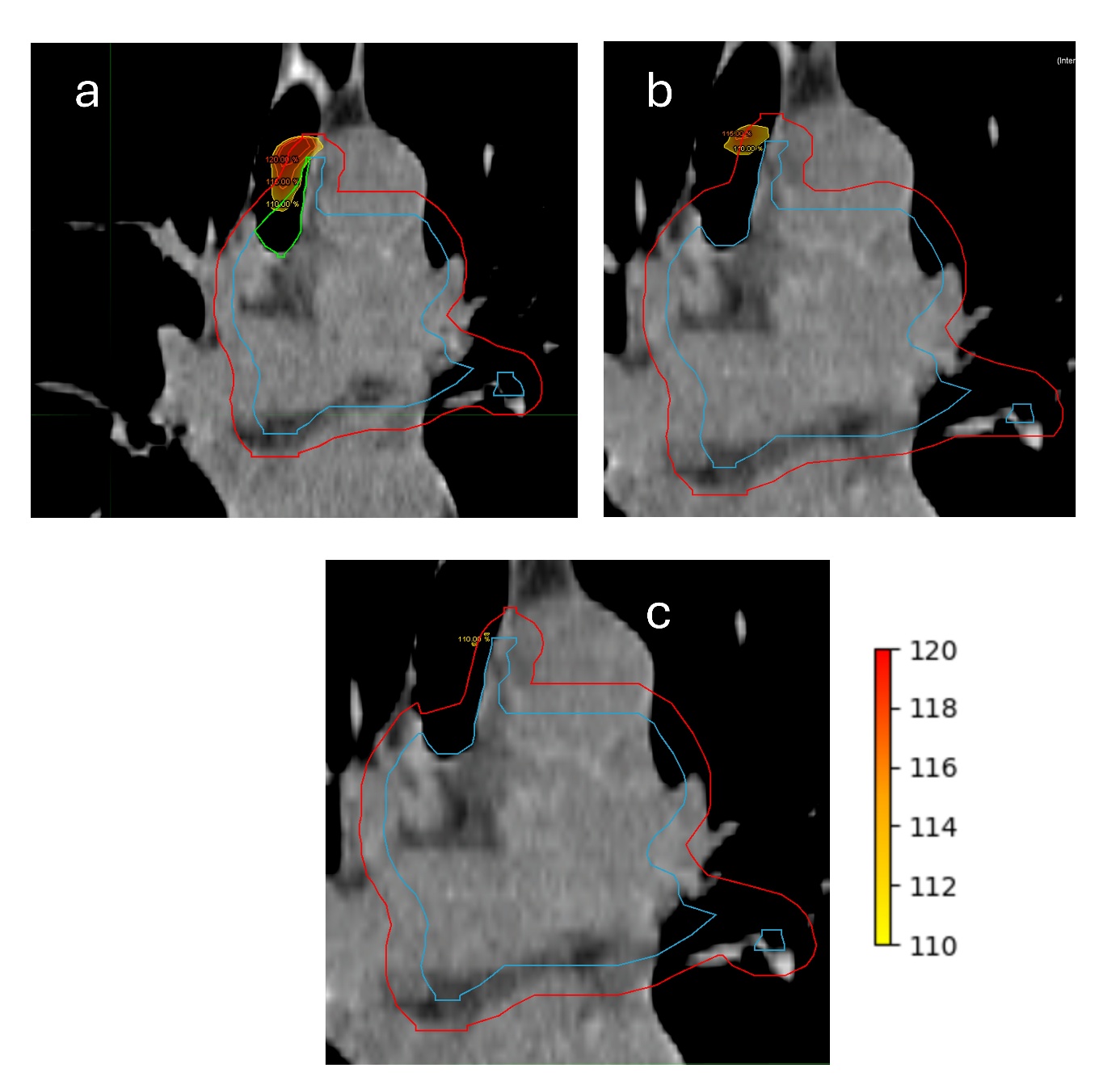


Suppl Figure 4: Dose distribution by the three methods S1, S2 and S3 used for treatment plan optimization characterized in the images a, b and c respectively. The dose distributions were calculated on CTplan by Acuros XB using water override of air in CBAL. Blue lines indicate the boundaries of the CTV, green lines those of the CTV-supplement in CBAL according to method S1, and red lines those of the PTV. Note, that the PTV differ between a, b, and c. The green line characterizes the helper CTV used for the generation of the PTV by method 1 depicted in panel a.
